# Construction and Validation of a Systematic Ethogram of *Macaca fascicularis* in a Free Enclosure

**DOI:** 10.1371/journal.pone.0037486

**Published:** 2012-05-25

**Authors:** Fan Xu, Liang Xie, Xin Li, Qi Li, Tao Wang, Yongjia Ji, Fei Kong, Qunlin Zhan, Ke Cheng, Liang Fang, Peng Xie

**Affiliations:** 1 Institute of Neuroscience, Chongqing Medical University, Chongqing, China; 2 Chongqing Key Laboratory of Neurobiology, Chongqing, China; 3 Department of Neurology, The First Affiliated Hospital of Chongqing Medical University, Chongqing, China; 4 Department of Neurology, The Fifth People's Hospital of Chongqing, Chongqing, China; Cajal Institute, Consejo Superior de Investigaciones Científicas, Spain

## Abstract

Behavioral studies in non-human primates have become ideal models for further investigations into advanced cognitive function in humans. To date, there is no systematic ethogram of the cynomolgus monkey (*Macaca fascicularis)* in a free enclosure. In a field observation of 6012 subjects, 107 distinct behaviors of *M. fascicularis* were preliminarily described. 83 of these behaviors were then independently validated through a randomized cohort and classified into 12 behavioral categories. 53 of these behaviors were then selected to accurately reflect the daily mundane activity of the species in a free enclosure. These findings systematically document the behavior of *M. fascicularis* in a free enclosure for use in further investigations.

## Introduction

Behavioral science has garnered considerable attention in contemporary neurological and psychological scientific circles as a platform for studying advanced cognitive function in humans. The United States declared 2000–2010 as the “Decade of Behavior” [1], in line with the increasing volume of research into psychopathology and human behavior. This phenomenon has been accompanied by a rising number of behavioral studies on human subjects [2].

However, due to ethical considerations, certain advanced functional and structural investigations cannot be conducted in humans [3], [4], [5], [6], [7], [8], [9], [10], [11], [12], [13], [14], [15]. Non-human primate models offer a viable alternative, as humans share neuroanatomical and psychological homology with these species. But, behavioral descriptions and classifications on non-human primates are often incomplete and scattered across disparate studies. Therefore, to support future behavioral investigations and concomitant quantitative studies, it is imperative to establish systematic ethogram of non-human primates on a species-specific basis [16], [17].

Several macaque species have demonstrated promise as non-human primate models for behavioral studies. In the 1930's, Skinner *et al.* [18] constructed a systematic ethogram of a captive lion-tailed macaque (*Macaca silenu*) through behavioral descriptions, definitions and classifications, as well as comparisons with other monkey species. Adams *et al.* [19] studied the sexual behavior of the adult cynomolgus monkey (*Macaca fascicularis*), uncovering the value of this macaque species as an animal model for human behavioral studies. Jolly *et al.* [20] reported this species is capable of acquiring feeding and social behaviors by example, indicating its behavioral homology to humans. William *et al.* [21] cataloged the behavior of 8 male rhesus monkeys (*Macaca mulatta*) for approximately 3 years under conditions of separation and confinement. Furthermore, Zhang, J. [22] investigated the post conflict settlement of group-living primate, among adult females Sichuan snub-nosed monkeys Rhinopithecus roxellana, which is crucial understanding for primate's competition and cooperation, and indicated that the pattern of post conflict affiliation demonstrates that the R. roxellana belongs to a tolerant species. In addition, Mear and Harlow [23] investigated the development of play behaviors in 8 rhesus infants over a 12-week period. Ultimately, Yamada, Kazunori [24] reviewed the studies concerning the social behavior of Old World monkeys and concluded that the social environments plays an important role in their daily routine on how to manage themselves.

However, to date, no systematic ethogram of the cynomolgus monkey (*M. fascicularis)* has been established. In this study, a comprehensive systematic ethogram (including behavioral descriptions, definitions and classifications) for *M. fascicularis* in a free enclosure was constructed and validated which satisfies the technical requirements for future hypothesis-testing and quantitative studies on the species.

## Results

**Figure 1:**
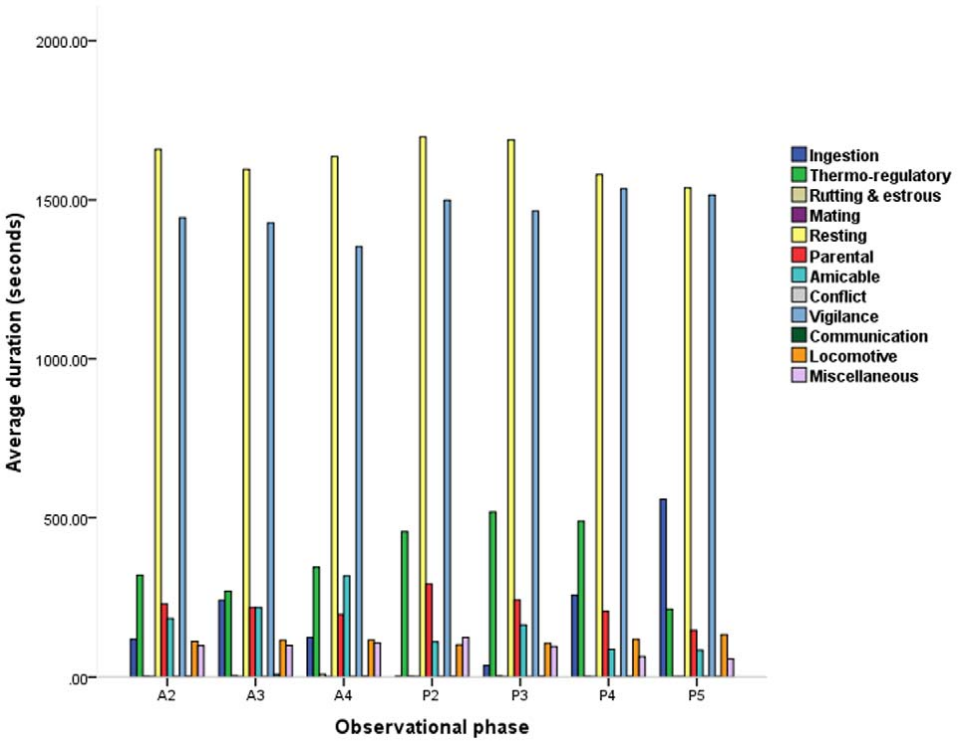
Average durations of 12 behavioral categories by observational phase. The average daily duration per subject (total duration of each behavior/40 subjects/4 days) are aggregated by category and displayed by phase. Phase timing: A2 = 10:00–10:30, A3 = 10:30–11:00, A4 = 11:00–11:30, P2 = 14:30–15:00, P3 = 15:00–15:30, P4 = 15:30–16:00, P5 = 16:00–16:30.

From April to December 2010, 107 behavioral items were created based on the Posture-Action-Environment principle [25], and the scanning method was employed to verify these behaviors among 6012 *M. fascicularis* subjects in a free enclosure. 83 of these behaviors were then validated on 40 randomly selected female subjects and classified into 12 behavioral categories .From this set of 83 behaviors, 53 behaviors were then selected from these 12 behavioral categories: i) ingestion, ii) thermo-regulatory, iii) rutting and estrous, iv) mating, v) resting, vi) parental, vii) amicable, iix) conflict, ix) vigilance, x) communication, xi) locomotive and xii) miscellaneous behaviors, more detail data, please see File S3. Across all 12 behavioral categories per day and 12 behavior categories within all observational phases per day, resting and vigilance behaviors occurred at a significantly greater average daily duration per subject than other behaviors (11.56× and 10.39× the median value, respectively; Fig. 1, Table 1 and table 2). Across all 53 behaviors and all observational phases, watching company (a vigilance behavior), quadrupedal walking on floor (a locomotive behavior), and sitting on floor (a resting behavior) were the most frequent behaviors observed (77.10×, 47.88×, and 46.27× the median value, respectively; Table 3). The average durations per subject for all 12 behavioral categories on both a daily and observational phase basis are listed in Tables 1 and 2, respectively. The average counts per subject for all 53 behaviors on both a daily and observational phase basis are detailed in Tables 3 and 4, respectively.

**Table 1:** The average daily duration per subject and associated multiples of the median value for all 12 behavioral categories.

Behavioral Category	N	Average Daily Duration per Subject (sec)	Multiple of Median Value
Ingestion	40	1340.57	1.36
Thermo-regulatory	40	2612.77	2.65
Rutting and estrous	40	26.11	0.03
Mating	40	5.65	0.01
Resting	40	11389.92	11.56
Parental	40	1535.95	1.56
Amicable	40	1167.14	1.18
Conflict	40	6.56	0.01
Vigilance	40	10237.72	10.39
Locomotive	40	803.16	0.82
Communication	40	20.93	0.02
Miscellaneous	40	648.04	0.66

Note: the median value of the average daily duration per subject calculated across all 12 behavioral categories is 985.15 seconds. The median was used as the measure of central tendency, as the average daily durations per subject are not normally distributed.

**Table 2:** Average daily duration per subject of all 12 behavioral categories segregated by observational phase.

Behavioral Categories	Average Daily Duration per Subject by Phase (sec)							Daily Total	
	N	A2	A3	A4	P2	P3	P4	P5	
Ingestion	40	119.21	241.74	124.83	2.58	35.80	258.38	558.03	1340.57
Thermo-regulatory	40	320.67	270.47	344.35	456.20	518.72	489.05	213.32	2612.78
Rutting and estrous	40	2.03	4.84	9.06	2.96	4.19	1.85	1.19	26.12
Mating	40	1.07	0.74	0.76	0.89	0.69	0.42	1.07	5.64
Resting	40	1658.03	1595.20	1635.99	1696.73	1688.00	1578.88	1537.10	11389.93
Parental	40	230.28	218.76	197.12	292.85	242.72	206.87	147.35	1535.95
Amicable	40	184.31	218.69	317.97	110.58	163.78	87.30	84.52	1167.15
Conflict	40	0.99	0.78	1.02	1.09	0.41	0.99	1.27	6.55
Vigilance	40	1444.19	1427.77	1352.86	1499.12	1465.13	1534.13	1514.53	10237.73
Locomotive	40	112.02	115.53	116.18	101.41	106.02	118.60	133.41	803.17
Communication	40	2.85	8.85	1.97	1.73	2.51	1.69	1.32	20.92
Miscellaneous	40	98.82	98.98	107.26	124.85	96.27	64.34	57.53	648.05

Note: A2 = 10:00–10:30, A3 = 10:30–11:00, A4 = 11:00–11:30, P2 = 14:30–15:00, P3 = 15:00–15:30, P4 = 15:30–16:00, P5 = 16:00–16:30.

**Table 3:** The average daily counts per subject and associated multiples of the median value for all 53 behaviors.

Behavior	N	Average Daily Count per Subject	Multiple of Median Value
Feeding while hanging	40	0.52	0.26
Feeding while sitting	40	7.50	3.73
Drinking	40	2.66	1.32
Chewing	40	11.20	5.57
Licking residue from floor	40	0.45	0.22
Eating object from body	40	0.09	0.04
Picking remaining food	40	7.92	3.94
Feeding while perched	40	0.47	0.23
Embracing	40	11.74	5.84
Licking genital area	40	0.01	0.00
Presenting buttocks	40	1.22	0.61
Mounting	40	0.06	0.03
Copulating	40	0.93	0.46
Sitting on floor	40	93.00	46.27
Sitting on floor facing wall	40	0.72	0.36
Perching on shelf	40	14.01	6.97
Lying on floor	40	0.83	0.41
Hanging on window or door	40	13.75	6.84
Hanging on iron chain	40	0.92	0.46
Hanging on skylight	40	1.27	0.63
Sitting and sleeping	40	0.15	0.07
Hanging on ventilator	40	0.16	0.08
Nursing infant	40	3.64	1.81
Holding infant	40	15.89	7.91
Grooming	40	7.29	3.63
Being groomed	40	9.00	4.48
Driving	40	0.03	0.01
Attacking	40	0.02	0.01
Fleeing	40	0.54	0.27
Biting	40	0.01	0.00
Being attacked	40	0.56	0.28
Shifting position	40	21.32	10.61
Alarmed jumping	40	2.30	1.14
Watching company	40	154.98	77.1
Miscellaneous calling	40	0.02	0.01
Lip smacking	40	2.01	1.00
Galloping	40	0.63	0.31
Walking on shelf	40	10.25	5.10
Quadrupedal walking on floor	40	96.23	47.88
Climbing	40	27.01	13.44
Walking on iron chain	40	0.42	0.21
Walking on skylight	40	2.33	1.16
Standing	40	8.64	4.30
Shaking body	40	5.61	2.79
Playing	40	1.22	0.61
Licking hair	40	3.32	1.65
Scratching by hind leg	40	6.76	3.36
Scratching by foreleg	40	44.01	21.90
Yawning	40	5.58	2.78
Digging anus	40	2.73	1.36
Rubbing paw on floor	40	0.34	0.17
Licking tail	40	0.81	0.40
Shaking ID card	40	0.64	0.32

Note: the median value of the average daily duration per subject calculated across all 53 behaviors is 2.01 seconds. The median was used as the measure of central tendency, as the average daily durations per subject are not normally distributed.

**Table 4:** The average daily count per subject for all 53 behaviors segregated by observational phase.

		Average Daily Count per Subject by Phase							
Behavior	N	A2	A3	A4	P2	P3	P4	P5	Daily Total
Feeding while hanging	40	0.07	0.09	0.04	0.00	0.00	0.18	0.15	0.53
Feeding while sitting	40	0.93	1.51	0.83	0.03	0.28	1.83	2.11	7.52
Drinking	40	0.13	0.18	0.16	0.14	0.18	0.50	1.38	2.67
Chewing	40	0.69	2.03	1.33	0.00	0.22	1.97	4.96	11.20
Licking residue from floor	40	0.07	0.05	0.09	0.04	0.04	0.08	0.08	0.45
Eating object from body	40	0.06	0.00	0.01	0.01	0.00	0.01	0.00	0.09
Picking remaining food	40	1.03	1.55	1.21	0.04	0.26	1.32	2.51	7.92
Feeding while perched	40	0.03	0.08	0.05	0.00	0.03	0.18	0.12	0.49
Embracing	40	1.42	1.40	1.55	1.90	2.19	2.08	1.21	11.75
Licking genital area	40	0.00	0.00	0.00	0.00	0.01	0.01	0.00	0.02
Presenting buttocks	40	0.11	0.21	0.36	0.19	0.17	0.09	0.09	1.22
Mounting	40	0.01	0.01	0.00	0.03	0.00	0.00	0.02	0.07
Copulating	40	0.16	0.12	0.12	0.14	0.10	0.11	0.18	0.93
Sitting on floor	40	12.44	13.95	14.21	11.82	12.09	13.68	14.81	93.00
Sitting on floor facing wall	40	0.06	0.11	0.13	0.06	0.10	0.17	0.09	0.72
Perching on shelf	40	1.98	1.76	1.95	1.96	2.16	2.00	2.21	14.02
Lying on floor	40	0.11	0.17	0.35	0.06	0.10	0.03	0.01	0.83
Hanging on window or door	40	1.71	2.34	1.72	1.82	1.71	1.98	2.48	13.76
Hanging on iron chain	40	0.18	0.13	0.11	0.18	0.14	0.11	0.08	0.93
Hanging on skylight	40	0.20	0.14	0.17	0.15	0.14	0.34	0.13	1.27
Sitting and sleeping	40	0.05	0.03	0.02	0.04	0.01	0.00	0.00	0.15
Hanging on ventilator	40	0.01	0.03	0.03	0.03	0.01	0.04	0.01	0.16
Nursing infant	40	0.75	0.81	0.51	0.71	0.46	0.19	0.21	3.64
Holding infant	40	2.14	2.16	1.78	2.93	2.44	2.60	1.84	15.89
Grooming	40	1.21	1.38	2.02	0.74	0.92	0.56	0.47	7.30
Being groomed	40	1.25	1.40	2.32	1.06	1.51	0.82	0.64	9.00
Driving	40	0.00	0.01	0.02	0.00	0.00	0.00	0.00	0.03
Attacking	40	0.00	0.00	0.00	0.02	0.00	0.00	0.00	0.02
Fleeing	40	0.15	0.09	0.09	0.05	0.06	0.04	0.05	0.02
Biting	40	0.01	0.00	0.01	0.00	0.00	0.00	0.00	0.02
Being attacked	40	0.03	0.07	0.11	0.10	0.03	0.07	0.16	0.57
Shifting position	40	2.99	3.23	3.11	2.50	2.46	2.98	4.06	21.33
Alarmed jumping	40	0.34	0.39	0.30	0.26	0.29	0.34	0.38	2.30
Watching company	40	21.03	23.70	23.69	19.69	20.74	22.76	23.38	154.99
Miscellaneous calling	40	0.00	0.01	0.00	0.00	0.00	0.01	0.00	0.02
Lip smacking	40	0.34	0.32	0.26	0.29	0.30	0.28	0.23	2.02
Galloping	40	0.09	0.09	0.09	0.06	0.10	0.08	0.13	0.64
Walking on shelf	40	1.42	1.35	1.24	1.36	1.56	1.58	1.74	10.25
Quadrupedal walking on floor	40	12.76	14.45	13.88	11.75	11.94	15.08	16.38	96.24
Climbing	40	3.44	4.43	3.60	3.56	3.28	4.06	4.64	27.01
Walking on iron chain	40	0.09	0.08	0.06	0.06	0.06	0.06	0.02	0.43
Walking on skylight	40	0.33	0.24	0.28	0.40	0.35	0.48	0.26	2.34
Standing	40	1.12	1.50	1.42	0.86	1.25	1.23	1.27	8.65
Shaking body	40	1.29	1.04	0.81	0.61	0.69	0.52	0.67	5.63
Playing	40	0.17	0.09	0.18	0.08	0.24	0.25	0.21	1.22
Licking hair	40	0.46	0.43	0.51	0.76	0.48	0.41	0.28	3.33
Scratching by hind leg	40	0.66	0.59	1.01	1.01	1.11	1.26	1.13	6.77
Scratching by foreleg	40	5.76	6.32	6.81	7.08	6.88	5.80	5.36	44.01
Yawning	40	1.01	0.66	0.72	1.00	1.13	0.77	0.28	5.57
Digging anus	40	0.15	0.33	0.38	0.51	0.39	0.46	0.51	2.73
Rubbing paw on floor	40	0.02	0.04	0.00	0.13	0.10	0.04	0.01	0.34
Licking tail	40	0.10	0.14	0.19	0.11	0.09	0.09	0.09	0.81
Shaking ID card	40	0.09	0.16	0.04	0.08	0.14	0.07	0.07	0.65

Note: A2 = 10:00–10:30, A3 = 10:30–11:00, A4 = 11:00–11:30, P2 = 14:30–15:00, P3 = 15:00–15:30, P4 = 15:30–16:00, P5 = 16:00–16:30.

**Figure 2:**
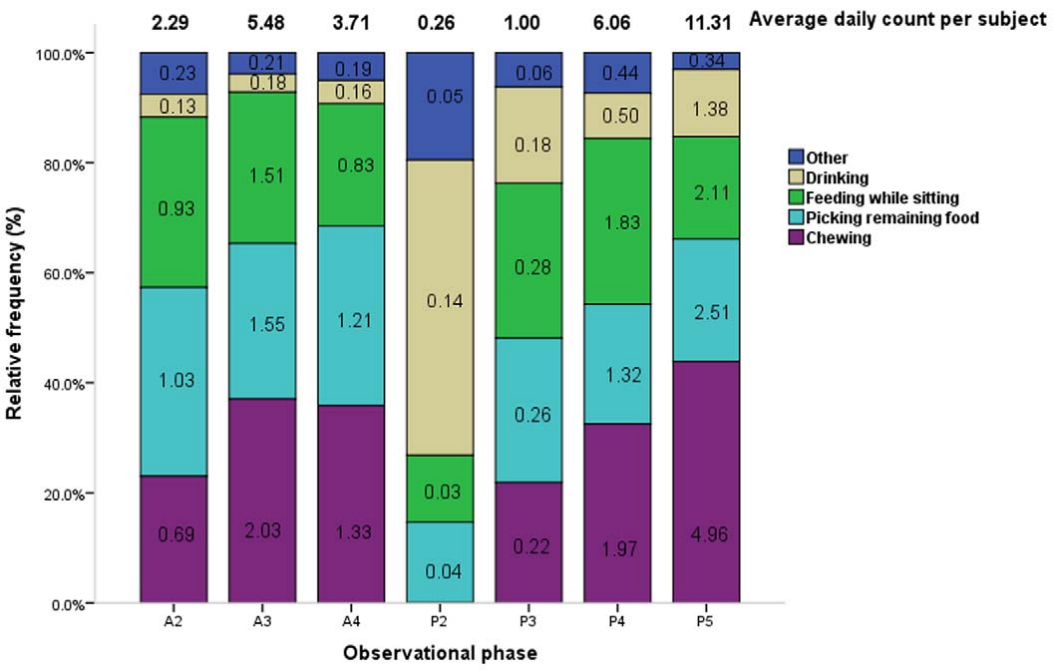
Average counts and relative frequencies of ingestion behaviors by observational phase. The average daily count per subject (total no. of actions/40 subjects/4 days) and relative frequencies of each ingestion behavior are displayed by phase. Each figure in related color bar represents the average daily frequencies of respective action per subject in each phase (total no. of actions/40 subjects/4 days).Phase timing: A2 = 10:00–10:30, A3 = 10:30–11:00, A4 = 11:00–11:30, P2 = 14:30–15:00, P3 = 15:00–15:30, P4 = 15:30–16:00, P5 = 16:00–16:30.

Ingestion behaviors occurred at a moderately higher average daily duration per subject with respect to other behavioral categories (1.36× the median value; Table 1). Chewing (5.57× the median value), picking remaining food (3.94× the median value), and feeding while sitting (3.73× the median value) were the most frequent ingestion behaviors observed across all phases with the notable exception of phase P2 (Fig. 2, Table 3) ingestion. In this phase, drinking (1.32× the median value) was the most frequently observed ingestion behavior. In addition, the average daily count per subject for ingestion behaviors was significantly higher in phase P5.

**Figure 3:**
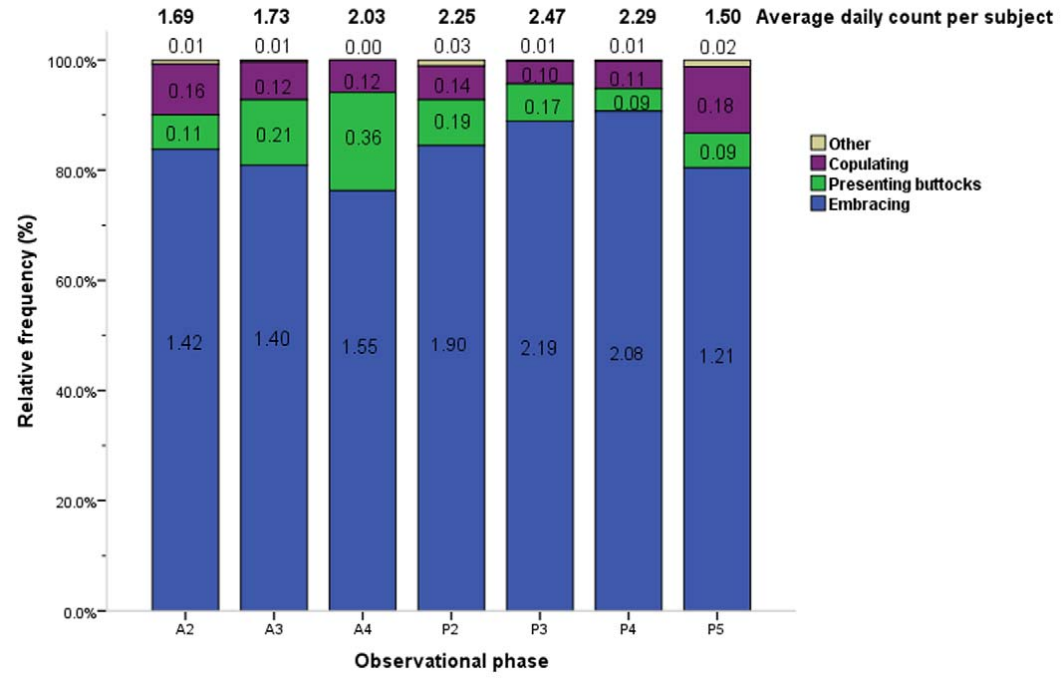
Average counts and relative frequencies of thermo-regulatory, rutting, estrous and mating behaviors by observational phase. The average daily count per subject (total no. of actions/40 subjects/4 days) and relative frequencies of each thermo-regulatory, rutting, estrous, and mating behavior are displayed by phase. Each figure in related color bar represents the average daily frequencies of respective action per subject in each phase (total no. of actions/40 subjects/4 days). Phase timing: A2 = 10:00–10:30, A3 = 10:30–11:00, A4 = 11:00–11:30, P2 = 14:30–15:00, P3 = 15:00–15:30, P4 = 15:30–16:00, P5 = 16:00–16:30.

Thermo-regulatory behavior occurred at a higher average daily duration per subject as compared to other behavioral categories (2.65× the median value; Table 1). Rutting, estrous and mating behaviors occurred at a significantly lower average daily duration per subject relative to other behavioral categories (0.03× and 0.01× the median value, respectively; Table 1). Average daily counts per subject and relative frequencies for thermo-regulatory, rutting and estrous, and mating behaviors are shown in Figure 3. Embracing, a thermo-regulatory behavior, accounted for the highest proportion of these behaviors across all phases (5.84× the median value). Presenting buttocks was the most frequent rutting and estrous behavior (0.61× the median value), and copulating (0.46× the median value) was the most frequent mating behavior across all phases. (Fig. 3, Table 3).

**Figure 4:**
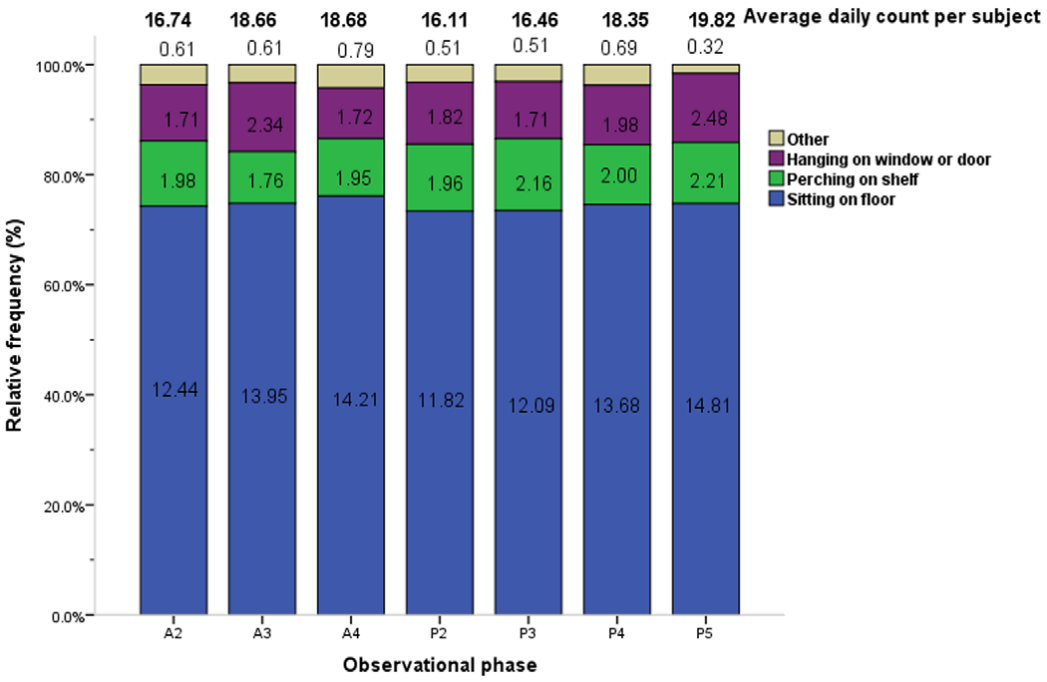
Average counts and relative frequencies of resting behaviors by observational phase. The average daily count per subject (total no. of actions/40 subjects/4 days) and relative frequencies of each resting behavior are displayed by phase. Each figure in related color bar represents the average daily frequencies of respective action per subject in each phase (total no. of actions/40 subjects/4 days). Phase timing: A2 = 10:00–10:30, A3 = 10:30–11:00, A4 = 11:00–11:30, P2 = 14:30–15:00, P3 = 15:00–15:30, P4 = 15:30–16:00, P5 = 16:00–16:30.

Resting behavior, the behavioral category of longest duration, occurred at a significantly higher average daily duration per subject as compared to other categories (11.56× the median value; Table 1). Across all phases, sitting on the floor (46.27× the median value) was the most frequent resting behavior, distantly followed by perching on shelf (6.97× the median value)shelf, and hanging on window or door (6.84× the median value; Fig. 4, Table 3).

**Figure 5:**
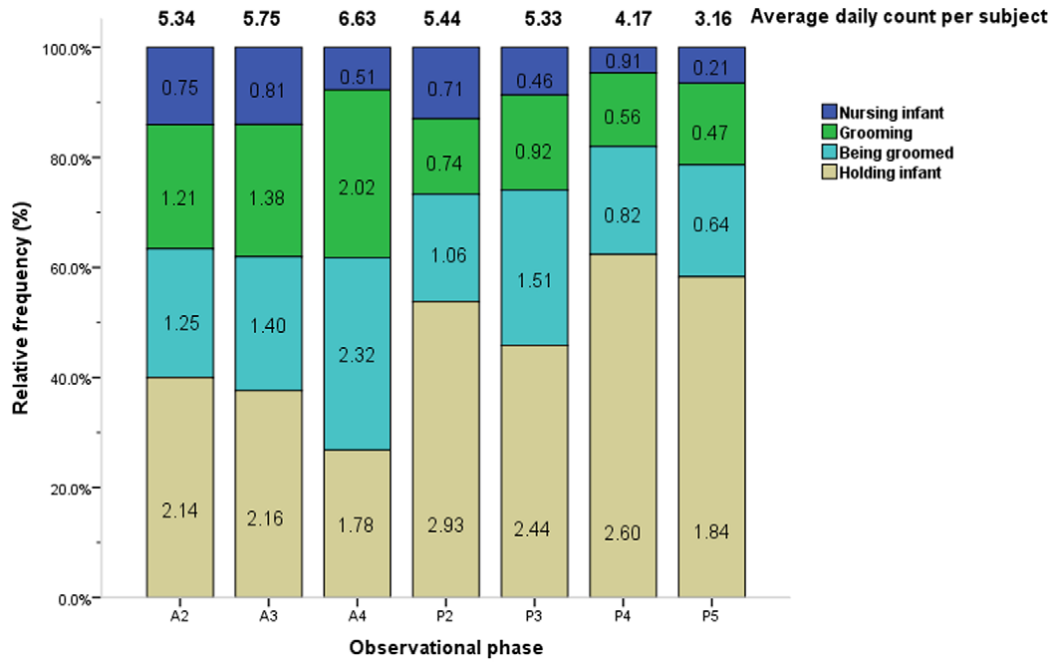
Average counts and relative frequencies of parental and amicable behaviors by observational phase. The average daily count per subject (total no. of actions/40 subjects/4 days) and relative frequencies of each parental and amicable behavior are displayed by phase. Each figure in related color bar represents the average daily frequencies of respective action per subject in each phase (total no. of actions/40 subjects/4 days). Phase timing: A2 = 10:00–10:30, A3 = 10:30–11:00, A4 = 11:00–11:30, P2 = 14:30–15:00, P3 = 15:00–15:30, P4 = 15:30–16:00, P5 = 16:00–16:30.

Parental and amicable behaviors occurred at a moderately higher average daily duration per subject as compared to other categories (1.56× and 1.18× the median value, respectively; Table 1). Among parental and amicable behaviors, four (4) primary constituent behaviors occurred in every phase. Across all phases, the frequencies of grooming and being groomed were approximately equivalent (3.63× and 4.48× the median value, respectively), and holding infant occurred approximately four (4) times more frequently than nursing infant (7.91× and 1.81× the median value, respectively; Fig. 5, Table 3).

**Figure 6:**
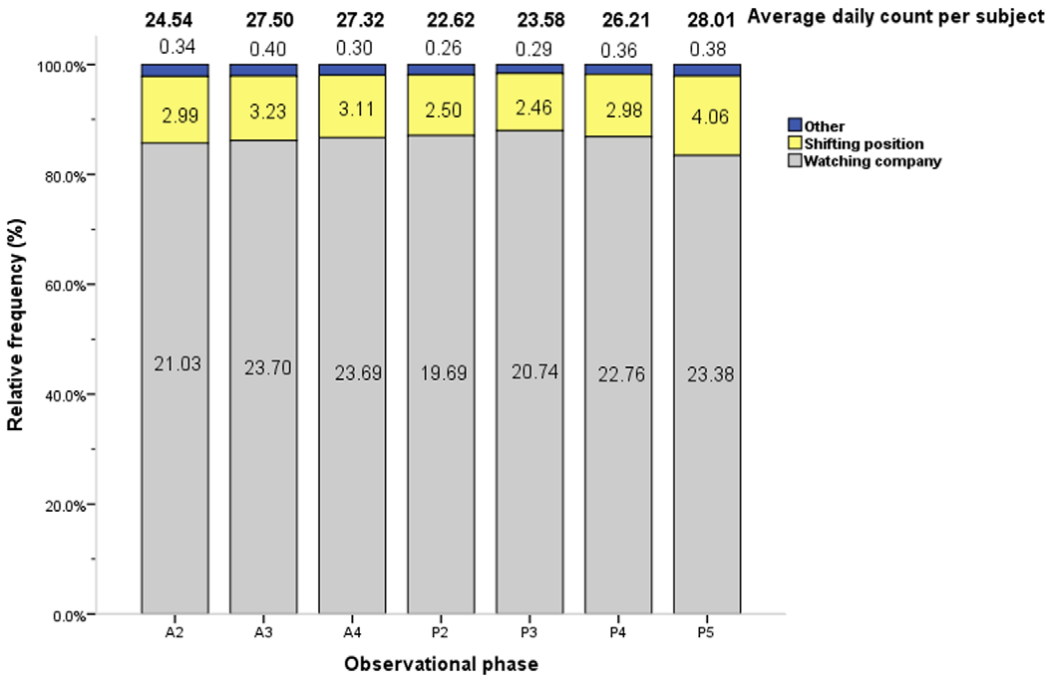
Average counts and relative frequencies of conflict and vigilance behaviors by observational phase. The average daily count per subject (total no. of actions/40 subjects/4 days) and relative frequencies of each conflict and vigilance behavior are displayed by phase. Each figure in related color bar represents the average daily frequencies of respective action per subject in each phase(total no. of actions/40 subjects/4 days). Phase timing: A2 = 10:00–10:30, A3 = 10:30–11:00, A4 = 11:00–11:30, P2 = 14:30–15:00, P3 = 15:00–15:30, P4 = 15:30–16:00, P5 = 16:00–16:30.

Conflict behaviors occurred at a significantly lower average daily duration per subject as compared to other categories; vigilance behaviors, in contrast, occurred at a significantly higher average daily duration per subject as compared to other categories (0.01× and 10.39× the median value, respectively; Table 1). Among conflict and vigilance behaviors across all phases, watching company was the most frequent behavior (77.10× the median value), distantly seconded by shifting position (10.61× the median value; Fig. 6, Table 3).

**Figure 7:**
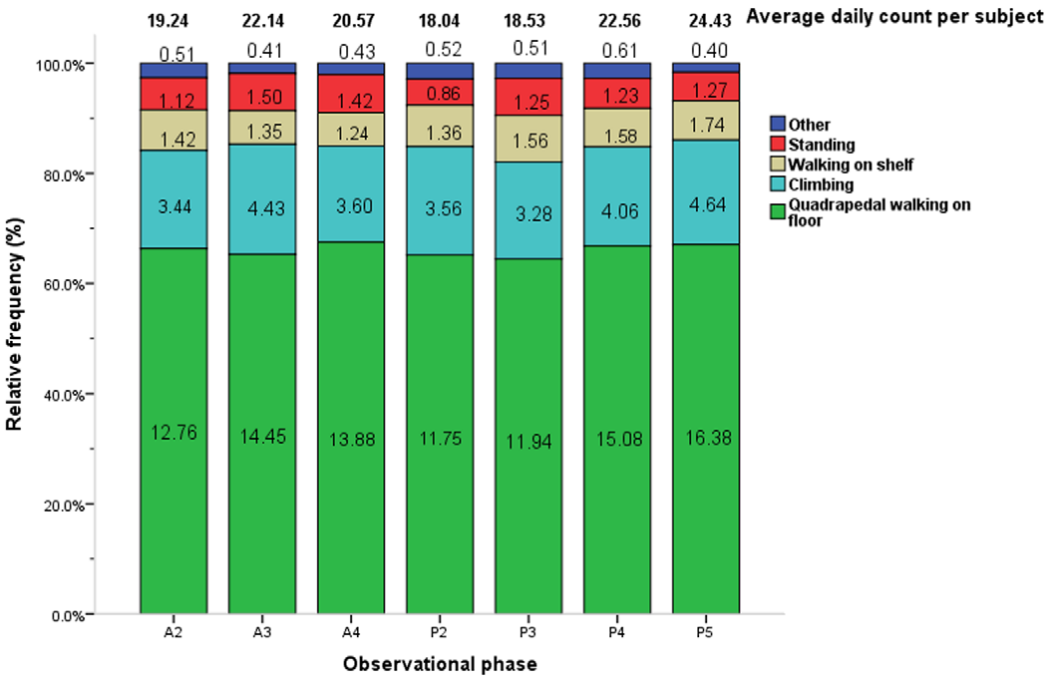
Average counts and relative frequencies of locomotive behaviors by observational phase. The average daily count per subject (total no. of actions/40 subjects/4 days) and relative frequencies of each locomotive behavior are displayed by phase. Each figure in related color bar represents the average daily frequencies of respective action per subject in each phase (total no. of actions/40 subjects/4 days). Phase timing: A2 = 10:00–10:30, A3 = 10:30–11:00, A4 = 11:00–11:30, P2 = 14:30–15:00, P3 = 15:00–15:30, P4 = 15:30–16:00, P5 = 16:00–16:30.

Locomotive behaviors occurred at a moderately lower average daily duration per subject as compared to other categories (0.82× the median value; Table 1). Among locomotive behaviors across all phases, quadrupedal walking on floor (47.88× the median value), climbing (13.44× the median value), walking on shelf (5.10× the median value) , and standing (4.30× the median value) were the most frequent behaviors (Fig. 7, Table 3).

**Figure 8:**
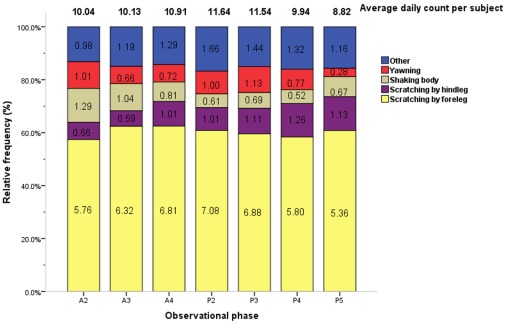
Average counts and relative frequencies of communication and miscellaneous behaviors by observational phase. The average daily count per subject (total no. of actions/40 subjects/4 days) and relative frequencies of each communication and miscellaneous behavior are displayed by phase. Each figure in related color bar represents the average daily frequencies of respective action per subject in each phase(total no. of actions/40 subjects/4 days).Phase timing: A2 = 10:00–10:30, A3 = 10:30–11:00, A4 = 11:00–11:30, P2 = 14:30–15:00, P3 = 15:00–15:30, P4 = 15:30–16:00, P5 = 16:00–16:30.

Communication behaviors occurred at a significantly lower average daily duration per subject as compared to other categories; miscellaneous behaviors occurred at a moderately lower average daily duration per subject (0.02× and 0.66× the median value, respectively; Table 1). Among communication and miscellaneous behaviors, scratching body by foreleg (21.90× the median value) was the most frequent behavior, distantly followed by scratching by hind leg (3.36× the median value), shaking body (2.79× the median value), and yawning (2.78× the median value; Fig. 8, Table 3).

## Discussion

The systematic ethogram of *M. fascicularis* in a free enclosure precisely describes, defines and classifies the behaviors observed in the daily mundane routine of this species. The most important characteristic of this ethogram is the coding and transfer of raw video-based observations into digital data for further statistical analysis. By employing this methodology, the ethogram satisfies the technical requirements for future hypothesis-testing and quantitative studies [16], [17] by enabling NOLDUS software [26] to perform advanced statistical analysis on behavioral data. Another technical merit of this ethogram is that it can be updated in real-time through direct field observation and data importation into the software for later analysis.

In addition to these technical advantages, underlying psychological elements can be assessed through an analysis of the quantitative data on observable behaviors. For example, appetite can be measured by the frequency and duration of ingestion behavior; the social relationships between subjects can be analyzed by the frequency and duration of amicable, conflict and vigilance behaviors; sexual drive can be measured by the frequency and duration of rutting, estrous and mating behaviors; activity levels can be ascertained by the frequency and duration of rest and locomotive behaviors; and social interaction and affect can be studied through the frequency and duration of communication and miscellaneous behaviors.

In the current study, resting and vigilance behaviors were the most prominently occurring behavioral categories, with sitting on the floor and watching company dominating their respective categories; this finding did not vary significantly by observational phase. It can be reasonably surmised that this species dedicates substantial time to conserving energy through rest and promoting social ties/avoiding conflict through vigilance. As to ingestion behaviors, it should be noted that there were three (3) daily feedings at 7:30, 10:30 (phase A3) and 16:00 (phase P5). The more frequent drinking behavior, absence of chewing behavior, and significantly lower average daily count per subject value in phase P2 is likely attributable to this fixed feeding schedule. Moreover, during the 10:30 feeding, primarily fresh fruit was supplied; this dietary difference may explain the fact that the average daily count per subject value in phase A3 was less than half the corresponding value in phase P5. As to rutting, estrous, and mating behaviors, it appears that these behaviors peak in frequency in phases A3–A4 and P5; whether this phenomenon is correlated or just coincident with the feeding schedule remains an open question. As to parental and amicable behaviors, the average daily count per subject value peaks and grooming behaviors are more frequent in phase A3–A4; as a speculation, the late morning may mark a time period for social bonding in this species. As to conflict, vigilance and locomotive behaviors, the average daily count per subject value peaks in phases A3–A4 and P5. This finding may be correlated with the feeding schedule, as conflict, vigilance and locomotive behaviors may be heightened in order to protect feeding position and/or food from other monkeys. As to communication and miscellaneous behaviors, the average daily count per subject value peaks in phases P2–P3; this category is primarily concerned with self-directed behaviors such as self-scratching and shaking. The early afternoon hours may mark a time period for solitary self-caring activities in this species. There are several limitations to this study. First, while observation of *M. fascicularis* in the wild would have been preferable, issues of subject identification and repeat observation required the use of a free enclosure environment. Second, it should be noted that observations spanned daytime hours only (9:30–11:30 and 14:30–16:30); thus, nocturnal behaviors were not investigated nor recorded in this study. Third, parturition behavior was not observed nor recorded. Fourth, behaviors were classified according to specific ecological functions. As a result, the edges of some behavioral sets may overlap, which may lead to inaccuracies in behavioral classifications. Fifth, some less commonly occurring behaviors may have been overlooked through human error. Further behavioral studies on the species can address these limitations.

In conclusion, this systematic ethogram of the cynomolgus monkey (*M. fascicularis)* in a free enclosure provides a platform for future behavioral investigations and concomitant quantitative studies on this species. These studies, combined with others on non-human primates, should provide further insight into advanced cognitive function in humans.

## Methods

### Location

The *M. fascicularis* Feeding Base of Zhongke Experimental Animal Co., Ltd. (hereinafter “the company”) is located in Suzhou, P.R.C. at E 31°07′03″ to 31°07′06″, N 120°19′08″to 120°19′15″. The company imported the *M. fascicularis* subjects from Guangdong and Vietnam in 1990 and established a domestication and breeding base for these monkeys.

### Observation

First, the initial observations of 6012 *M. fascicularis* subjects followed by a detailed verification were performed by the scanning method to construct the preliminary ethogram. Then, an independent validation by focal observation was performed on a randomized cohort of 40 young adult female *M. fascicularis* subjects (each weighing 3–6 kg and aged 8–16 years)who were selected by means of simple random sample, seed 20101207, from the original population (n = 6012). The subjects were housed in 25 free enclosures measuring 8 m×3 m×3 m (L×W×H), given water and libitum, and fed daily with fresh fruit, vegetables and compound high-nutrition monkey food. Two (2) male and 20 female subjects were placed into each free enclosure to match the wild male: female ratio range of 1∶7–11. There was no statistically significant difference in male: female ratios across all free enclosures (chi square test, person = 1.0). All free enclosures were consistently maintained on a 12 h light/dark cycle.

In recording observations, three (3) high-pixel videos of the subjects were recorded using a SONY 1100 megapixel camcorder. Then, a Lenovo PC was used to transfer and convert the videos into a viewable format for analysis through NOLDUS Observer XT software (version 10.0, Noldus Information Technology, Leesburg, PA) [26]. The list of behaviors was encoded to satisfy the operational requirements of the software [27]. Three (3) qualified observers blindly watched the videos and used the software to record data on the frequency and duration of each behavior. Inter-observer reliability between the three (3) observers was determined to be greater than 85% for each behavior.

A six (6)-month preliminary observation was performed from April-December 2010. More than 1000 hours of video footage was collected to obtain a comprehensive view of the subjects' behavior. A systematic ethogram was then constructed using behavioral observations collected from qualitative sampling *ad libitum*, focal animal sampling and instantaneous scans. Sampling was limited to daytime hours after feeders completed their daily cleaning procedure (9:30–11:30 and 14:30–16:30 daily).

### Ethics Statement

All procedures described were observational under normal rearing circumstance and did not involve physical manipulation of the subjects or changes to their environment or diet. Animal care and housing procedures were in compliance with Chinese regulatory requirements (see addendum entitled “Ethics Statement on Non-human Primate Research”) and AAALAC statements.

In brief, complete animal husbandry and veterinary care was provided daily. Animals were fed a nutritious standardized diet, supplemented daily with fresh fruits and vegetables. Animals had unrestricted access to potable water. Animal enclosures were cleaned daily. Animals were observed daily by trained care-takers. Any observed abnormality, disease or injury was reported to the veterinary staff for diagnosis and treatment; this veterinary support was documented in both hardcopy and electronic formats.

In addition, this study was performed in strict accordance with the recommendations in the Guide for the Care and Use of Laboratory Animals of the Institute of Neuroscience of Chongqing Medical University (Approval No: 20100031). The protocol was approved prior to implementation by the Committee on the Ethics of Animal Experiments at the Chongqing Medical University and is in accordance with state regulations.

### Definitions

Behavior is generally defined as a “combination of posture and action, with obvious environmental adaptation functions.” Each discreet behavior occurs at a location within the environment. Therefore, the behavior of *M. fascicularis* in a free enclosure environment can be segregated into three discreet elements: posture, action and location. [25] These elements are defined as follows:

Posture: a particular bearing of the subject's body.
Action: a coordinated series of contractions, extensions, rotations and/or translations of the subject's body part(s) occurring in a relatively short period of time.
Location: a specific position within the free enclosure environment.

### Ethogram Construction

#### Posture Coding

Through observation, nine (9) postures were identified and encoded. These postures can be separated into two (2) groups: static postures (standing, sitting, lying, mounting, hanging) and dynamic postures (walking, galloping, jumping, climbing).These postures are defined are defined as follows:

Standing: supporting the body erect on hind legs or all four limbs for at least 5 seconds
Sitting: resting on the buttocks.
Lying: resting recumbent on the floor or shelf.
Mounting: rising upon the rear of another subject for copulation.
Hanging: suspending on four limbs from the skylight.
Walking: translating by steps on the floor.
Galloping: translating swiftly such that all limbs leave the floor for an instant.
Jumping: springing up from the floor by the muscular contraction of the limbs.
Climbing: ascending by all limbs along the window or the door.

#### Action coding

All actions are encoded and defined in the attached addendum entitled “File S1 and File S2”.

#### Location coding

Seven (7) locations were identified and encoded: floor, skylight, iron chain, shelf, window, door, and ventilator.

#### Posture-Action-Location System

From April to December 2010, 107 behavioral items were created based on the Posture-Action-Environment principle, and the scanning method was employed to verify these behaviors among 6012 *M. fascicularis* subjects in a free enclosure. 83 of these behaviors were then independently validated and classified into 12 behavioral categories through focal observation of a randomized cohort of 40 young-adult female *M. fascicularis* subjects (each weighing 3–6 kg and aged 8–16 years) selected from the original population (n = 6012). 53 of these behaviors were then selected belonging to 12 categories based on ecological function: ingestion, thermo-regulatory, rutting and estrous, mating, resting, parental, amicable, conflict, vigilance, locomotive, communication and miscellaneous behaviors. These behavioral categories are defined below:

Ingestion behavior: taking food material into the subject's digestive system.
Thermo-regulatory behavior: regulating body heat through movement.
Rutting and estrous behavior: arousing sexual interest in another subject.
Mating behavior: sexual intercourse between opposite-sex subjects.
Resting behavior: refraining from activity.
Parental behavior: providing supervision and care to the subject's offspring.
Amicable behavior: providing friendly actions towards another subject, such as grooming.
Conflict behavior: struggling between subjects resulting from incompatible or opposing demands.
Vigilance behavior: responding to threatening external stimuli.
Locomotive behavior: engaging in activity (as opposed to resting behavior).
Communication behavior: transmitting information to other subjects via gestures, emitting sounds, etc.
Miscellaneous behavior: a set of defined miscellaneous actions.

## Supporting Information

File S1 **Behaviorial Patterns of M.F.**
(DOC)

File S2 **Behavioral Definitions and Ethogram Validation.**
(DOC)

File S3 **Ethogram-raw data.**
(XLS)
