# Supplementary material for: Construction and Validation of a Systematic Ethogram of Macaca fascicularis in a Free Enclosure
Source: PLoS One. 2012 May 25;7(5):e37486. doi: 10.1371/journal.pone.0037486 (PMC3360774; doi:10.1371/journal.pone.0037486)
Supplement: File S1 — Behaviorial Patterns of M.F. (DOC) [file pone.0037486.s001.doc]

**Behavioral Definitions and Ethogram Validation**

An ethogram classifying the behavioral characteristics of *Macaca fasciculari*s in a free enclosure was prepared. By employing the scanning method on a total population of 6012 subjects, 107 distinct behaviors were defined by standard descriptions; these have been classified and listed below in 14 behavioral categories. Then, 83 of these behaviors across 12 behavioral categories were validated in a randomized cohort of 40 female subjects for use in the ethogram; these are listed in the second section of this addendum. In addition, some behaviors have been described with attached photographs; these are located in the addendum entitled “The Behavior Patterns of Macaca fascicularis Reared in a Free Enclosure.”

**Behavioral Definitions for *M. fascicularis* in a Free Enclosure**

**A. Ingestion behaviors**

1. Searching: searching for food by hand, until food is acquired.

2. Feeding while squatting: eating food while squatting on the floor (not sitting).

3. Feeding while hanging: eating food while hanging from the skylight.

4. Feeding while sitting: eating food while sitting on the floor.

5. Drinking: imbibing water from the tube located on the wall.

6. Chewing: chewing food or any other substance placed into the mouth.

7. Licking residue from floor: licking of food residue scattered on the floor.

8. Eating object from body: eating object caught from body (e.g., insect, debris).

9. Picking remaining food: picking remaining food by hand that has been abandoned by other subjects.

10. Feeding while perched: eating food while perched on the shelf.

11. Suckling: self-suckling of subject's nipple.

**B. Elimination** **behaviors**

12. Urinating under duress: urinating while under duress from aggressor.

13. Urinating while hanging: urinating while hanging on the iron chain or skylight.

14. Urinating while lying: urinating while lying on the floor or shelf.

15. Urinating while climbing: urinating while climbing on the window or door.

16. Urinating while standing: urinating while standing on the floor.

17. Defecating while walking: defecating while walking on the floor.

18. Defecating while standing: defecating while standing on the floor.

19. Defecating under duress: defecating while under duress from aggressor.

20. Defecating while hanging: defecating while hanging on the iron chain or skylight.

21. Defecating while lying: defecating while lying on the floor or shelf.

22. Defecating while climbing: defecating while climbing on the window or door.

**C. Thermo-regulatory behaviors**

23. Huddling: huddling (self-embracing) on the floor.

24. Quivering: quivering on the floor.

25. Embracing: embracing or being embraced by another subject.

**D. Rutting and estrous behavior**

26. Licking genital area: licking genital region of another subject.

27. Presenting buttocks: presenting the buttocks to another subject.

28. Peri-vulvar discoloration: discoloration of the peri-vulvar area during estrous.

29. Sniffing ano-genital area: sniffing the ano-genital region of another subject.

30. Sniffing urine: sniffing another subject's urine.

31. Rolling tongue: rolling of the tongue across the lips.

32. Chasing: chasing another subject.

33. Homosexual roaring: roaring during courting (observed during homosexual encounters).

34. Homosexual mounting: pelvic mounting observed during homosexual encounters.

35. Tail arching: arching of the tail (observed in male subjects preparing to fight).

**E. Mating behavior**

36. Mounting: pelvic mounting observed during heterosexual encounters.

37. Copulating: sexual intercourse between heterosexual subjects.

38. Post-copulation guarding: guarding mate following copulation.

39. Post-copulation standing: standing stationary behind mate following copulation.

40. Ejaculating: ejaculating following masturbation.

41. Masturbating: self-manipulation of subject's penis.

**F. Parturition behavior**

42. Panning: searching for a suitable place to give birth.

43. Birthing contractions: birthing contractions experienced just prior to delivery.

44. Delivery: neonate exiting the birth canal.

45. Eating placenta: the mother eating the placenta

46. Licking genital region of infant: the mother licking the genital region of the infant.

47. Licking body of infant: the mother licking the body of the infant.

**G. Resting behavior**

48. Sitting on floor: sitting on the floor.

49. Sitting on floor facing wall: sitting on the floor while facing the wall.

50. Perching on shelf: perching on the shelf.

51. Sitting on ring: sitting on the ring that is hanging from the ceiling.

52. Lying on floor: lying on the floor.

53. Lying on shelf: lying on the shelf.

54. Hanging on window or door: hanging on the window or door.

55. Hanging on skylight: hanging on the skylight.

56. Hanging on iron chain: hinging on the iron chain that is hanging from the ceiling.

57. Hanging on ventilator: hanging on the ventilator.

58. Sitting and sleeping: Sleeping while seated.

**H. Parental behavior**

59. Nursing infant: the mother nursing the infant.

60. Holding infant: the mother holding the infant (including embracing and holding the infant by the tail).

61. Defending infant: the mother protecting the infant from aggressors (including embracing and retreating).

62. Licking anus of infant: the mother licking anus of the infant.

63. Checking anus of infant: the mother inspecting the anus of the infant.

**I. Amicable behavior**

64. Grooming: grooming another subject.

65. Being groomed: being grooming by another subject.

**J. Conflict behavior**

65. Driving: driving another subject away (when competing for food or a mate, etc.).

67. Attacking: physically attacking another subject (when competing for food or a mate, etc.).

68. Fleeing: fleeing from a fight.

69. Pulling foreleg: subject pulling another subject's foreleg.

70. Pulling hind leg: subject pulling another subject's hind legs.

71. Protracting ears: subject's ears protract forward (indicating hostility).

72. Threatening: threatening another subject (including staring, opening the mouth, and/or baring the teeth).

73. Being threatened: being threatened by another subject.

74. Being attacked: being attacked by another subject.

75. Parallel pacing: pacing in parallel with another subject.

76. Biting: Biting another subject.

**K. Vigilance behavior**

77. Shifting position: shifting position in response to a threat.

78. Alarmed jumping: jumping in alarm in response to a threat.

79. Watching company: keeping watch of other subjects.

80. Alarmed calling: calling an alarm to the group in response to a threat.

81. Miscellaneous calling: calling behavior not seemingly directed to another subject or to the group.

82. Shaking cage: shaking the cage.

**L. Locomotive behavior**

83. Galloping: galloping on the floor on all four limbs (faster rate than trotting).

84. Walking on shelf: walking on the shelf.

85. Quadrupedal walking on floor: walking on the floor on all four limbs.

86. Moving: moving to another location when approached by another.

87. Climbing: climbing on the window or door.

88. Walking on iron chain: walking on the iron chain by all four limbs.

89. Walking on skylight: walking on the skylight by all four limbs.

90. Standing: standing up on all 4 limbs on the floor or shelf.

91. Stepping: slow-stepping on the floor on all four limbs (slower rate than quadrupedal walking ).

92. Trotting: trotting on the floor on all four limbs (faster rate than quadrupedal walking).

**M. Communication behavior**

93. Lip smacking: smacking of the lips in communicating with another subject.

94. Sniffing: sniffing another subject's body.

95. Voiding: voiding urine to communicate with another subject.

**N. Miscellaneous behavior**

96. Shaking body: self-shaking of the subject's body (in order to remove objects from hair).

97. Playing: playing with a toy or an infant.

98. Licking hair: self-licking of the subject's hair.

99. Scratching by hind leg: self-scratching of the subject's body by the hind leg.

100. Scratching by foreleg: self-scratching of the subject's body by the foreleg.

101. Yawning: yawning.

102. Licking hand: self-licking of subject's hand.

103. Catching pest: catching a flying pest hovering around the subject.

104. Digging anus: self-digging into one's anus.

105. Rubbing palm on floor: rubbing the palm back and forth upon the floor.

106. Licking tail: self-licking of the subject's tail.

107. Shaking ID card: shaking the ID card placed around the subject's neck.

## Ethogram Validation

The 83 validated behaviors are checked off in the list below. The 53 bolded behaviors are those finally selected for data recording.

| **Ingestion behavior** | **Elimination behavior** |
| --- | --- |
| 1. Searching √  2. Feeding while squatting √  **3. Feeding while hanging √**  **4. Feeding while sitting √**  **5. Drinking √**  **6. Chewing √**  **7. Licking residue from floor √**  **8. Eating object from body √**  **9. Picking remaining food √**  **10. Feeding while perched √**  11. Suckling √ | 12. Urinating under duress √  13. Urinating while hanging √  14. Urinating while lying √  15. Urinating while climbing √  16. Urinating while standing √  17. Defecating while walking √  18. Defecating while standing √  19. Defecating under duress √  20. Defecating while hanging √  21. Defecating while lying **√**  22. Defecating while climbing **√** |
| **Thermo-regulatory behavior** | **Rutting and estrous behavior** |
| 23. Huddling √  24. Quivering √  **25. Embracing √** | **26. Licking genital area √**  **27. Presenting buttocks √**  *Peri-vulvar discoloration*  *Sniffing ano-genital area*  *Sniffing urine*  *Rolling tongue*  *Chasing*  *Homosexual roaring*  *Homosexual mounting*  *Tail arching* |
| **Mating behavior** | **Parturition behavior** |
| **28. Mounting √**  **29. Copulating √**  *Post-copulation guarding*  *Post-copulation standing*  *Ejaculating*  *Masturbating* | *Panning*  *Birthing contractions*  *Delivery*  *Eating placenta*  *Licking genital region of infant*  *Licking body of infant* |
| **Resting behavior** | **Parental behavior** |
| **30. Sitting on floor √**  **31. Sitting on floor facing wall √**  **32. Perching on shelf √**  33. Sitting on ring √  **34. Lying on floor √**  35. Lying on shelf √  **36. Hanging on window or door √**  **37. Hanging on skylight √**  **38. Hanging on iron chain √**  **39. Hanging on ventilator √**  **40. Sitting and sleeping √** | **41. Nursing infant √**  **42. Holding infant √**  43. Defending infant √  44. Licking anus of infant √  *Checking anus of infant* |
| **Amicable behavior** | **Conflict behavior** |
| **45.Grooming √**  **46. Being groomed √** | **47. Driving √**  **48. Attacking √**  **49. Fleeing √**  **50. Biting √**  51. Pulling foreleg √  52. Pulling hind leg √  53. Protracting ears √  54. Threatening √  55. Being threatened √  **56. Being attacked √**  *Parallel pacing* |
| **Vigilance behavior** | **Communication behavior** |
| **57. Shifting position √**  **58. Alarmed jumping √**  **59. Watching company √**  60. Alarmed calling √  **61. Miscellaneous calling √**  62. Shaking cage √ | **63. Lip smacking √**  *Sniffing*  *Voiding* |
| **Locomotion behavior** | **Miscellaneous behavior** |
| 64. Galloping √  **65. Walking on shelf √**  **66. Quadrupedal walking on floor √**  67. Moving √  **68. Climbing √**  **69. Walking on iron chain √**  **70. Walking on skylight √**  **71. Standing √**  *Stepping*  *Trotting* | **72. Shaking body √**  **73. Playing √**  **74. Licking hair √**  **75. Scratching by hind leg √**  **76. Scratching by foreleg √**  **77. Yawning √**  78. Licking hand √  79. Catching pest √  **80. Digging anus √**  **81. Rubbing palm on floor √**  **82. Licking tail √**  **83. Shaking ID card √** |
