# Supplementary material for: Construction and Validation of a Systematic Ethogram of Macaca fascicularis in a Free Enclosure
Source: PLoS One. 2012 May 25;7(5):e37486. doi: 10.1371/journal.pone.0037486 (PMC3360774; doi:10.1371/journal.pone.0037486)
Supplement: File S2 — Behavioral Definitions and Ethogram Validation. (DOC) [file pone.0037486.s002.doc]

**The Behavior Patterns of *Macaca fascicularis* Reared in a Free Enclosure**

# Behavior Coding System

| ***Macaca fascicularis*** | **14 Behavioral Categories** | | |
| --- | --- | --- | --- |
| 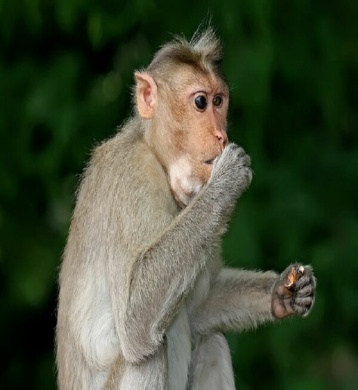 | A. Ingestion | B. Elimination | C. Thermo-regulatory |
|  | D. Rutting and estrous | E. Mating | F. Parturition |
|  | G. Resting | H. Parental | I. Amicable |
|  | J. Conflict | K. Vigilance | L. Locomotive |
|  | M. Communication | N. Miscellaneous |  |

| **Notice: The subject is *Macaca fascicularis* reared in free enclosure.** | | | | |
| --- | --- | --- | --- | --- |
| **A. Ingestion Behavior** | | | | |
| **Behavior** | | | | Searching |
| **Description** | | | | Searching for food (including edible debris, fruit, pills) on the floor, wall, or cage by hand. Typically, the subject sits and walks on the floor or on the door while searching. |
| 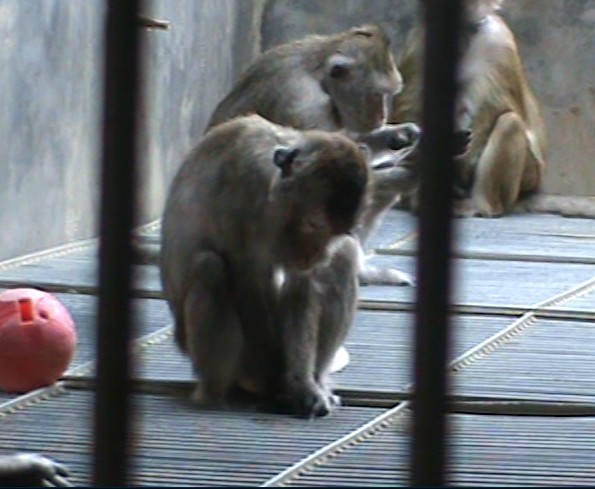 | | | | |
| **Behavior** | | | Feeding while squatting | |
| **Description** | | | Consuming food while squatting on the floor. Typically, the subject remains vigilant to avoid being invaded by other subjects of higher rank. | |
| 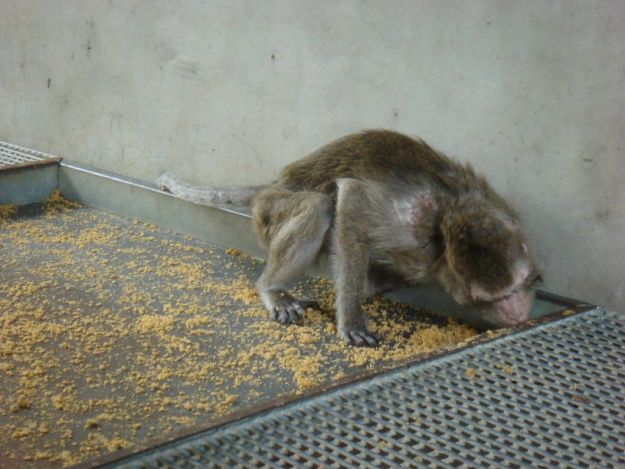 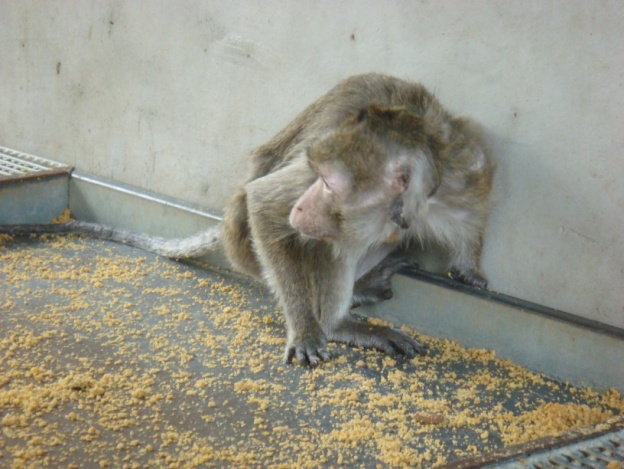 | | | | |
| **Behavior** | | Feeding while sitting | | |
| **Description** | | Consuming food while sitting on the floor. | | |
| 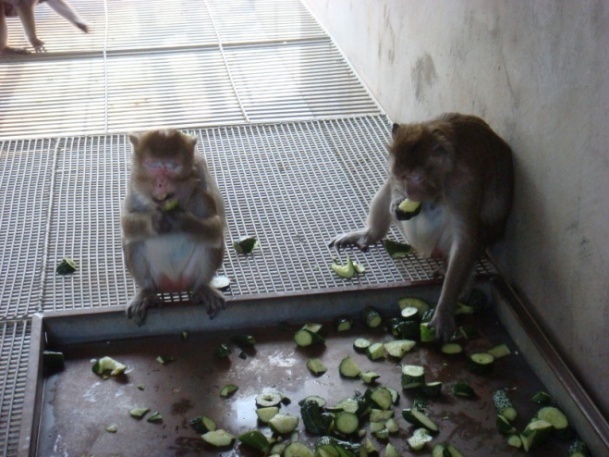 | | | | |
| **Behavior** | | | | Drinking |
| **Description** | | | | Drinking water from the tube embedded in the wall while sitting or standing. |
| 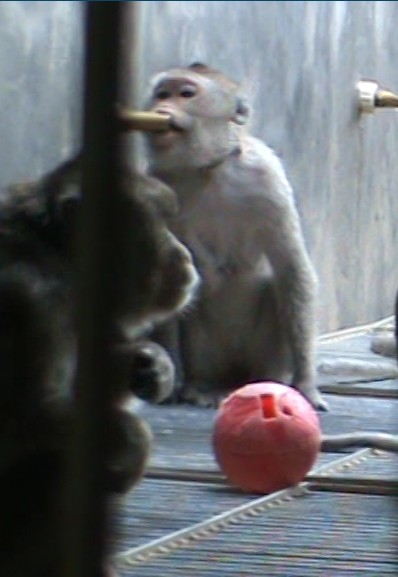 | | | | |
| **Behavior** | | | | Chewing |
| **Description** | | | | Chewing food while sitting, climbing or hanging. |
| 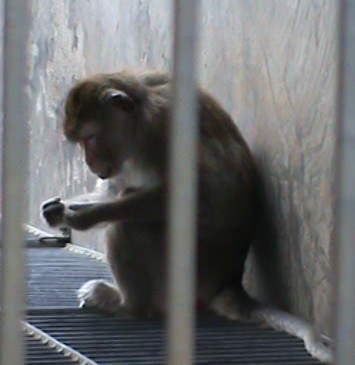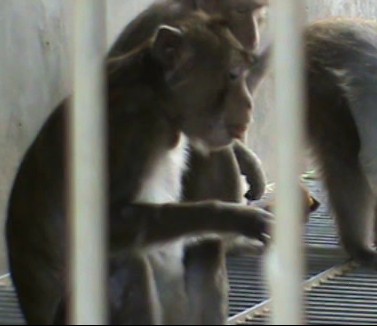 | | | | |
| **Behavior** | Suckling | | | |
| **Description** | Self-suckling on the nipple. | | | |
| 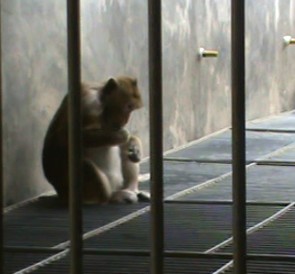 | | | | |
| **B. Elimination Behavior**  **This behavior was not validated.** | | | | |
| **C. Thermo-regulatory Behavior** | | | | |
| **Behavior** | | | | Embracing |
| **Description** | | | | Embracing others to retain body heat (typically occurs under low-temperature conditions). |

| **D. Rutting and Estrous Behavior** | |  |
| --- | --- | --- |
| **Behavior** | | Licking genital area |
| **Description** | | Licking the genital area of another subject, regardless of sex. |
| 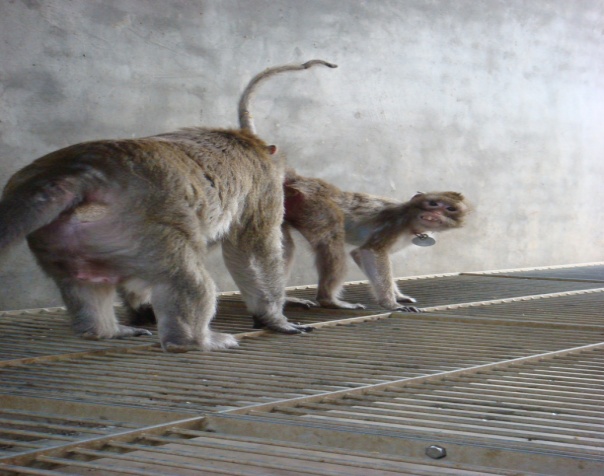 | | |
| **Behavior** | Presenting buttocks | |
| **Description** | Presenting the buttocks to another subject, regardless of sex (typically, a sign of post-conflict submission). | |
| 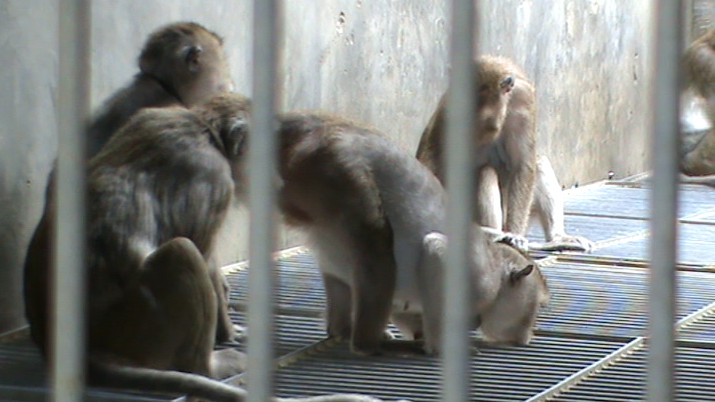 | | |

| **E. Mating Behavior** | | | |
| --- | --- | --- | --- |
| **Behavior** | | | Mounting |
| **Description** | | | A male subject mounting a female subject. |
| **Behavior** | | | Copulating |
| **Description** | | | A male subject mounting a female subject, then inserting the penis into the vagina accompanied by oscillating motion lasting for at least 3 seconds. |
| 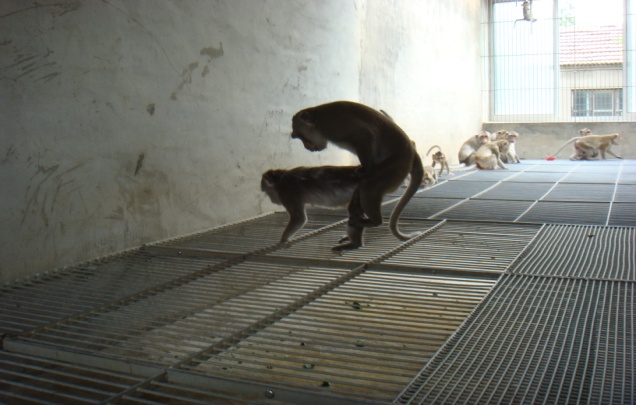  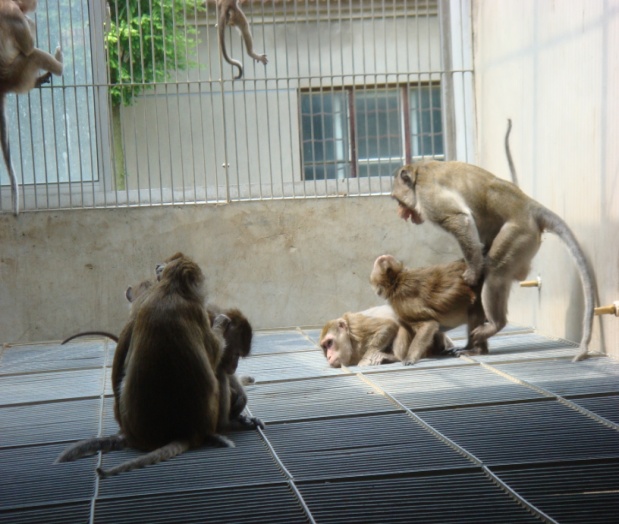 | | | |
| **F. Parturition Behavior**  **This behavior was not validated.** | | | |
| **G. Resting Behavior** | | | |
| **Behavior** | Sitting on floor | | |
| **Description** | Sitting on the floor to rest. | | |
| 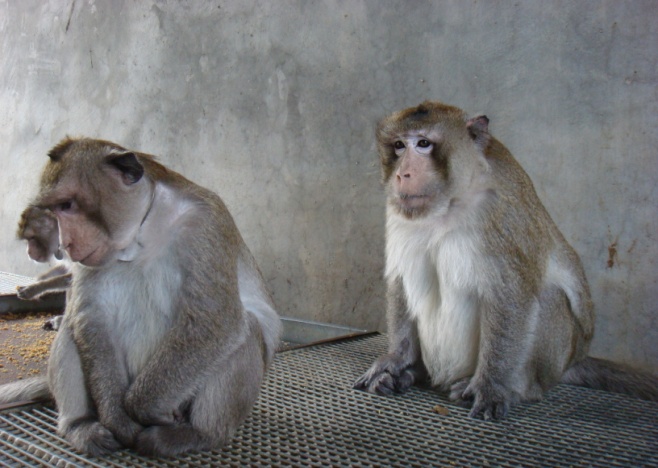 | | | |
| **Behavior** | Lying on floor | | |
| **Description** | Lying on the floor to rest. | | |
| 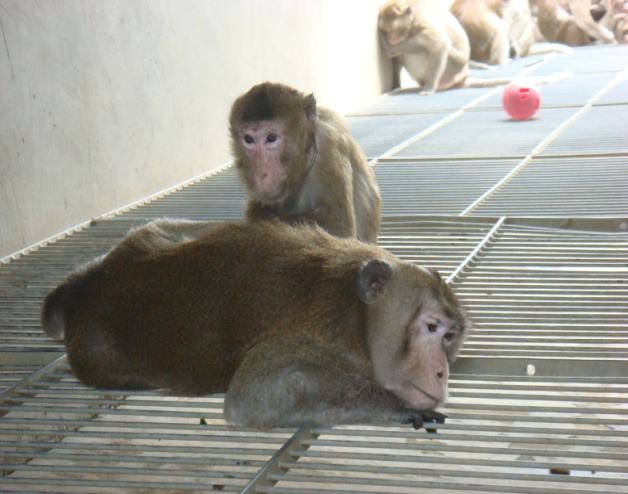 | | | |
| **Behavior** | | Hanging on door or window | |
| **Description** | | Hanging on the door or window to rest. | |
| 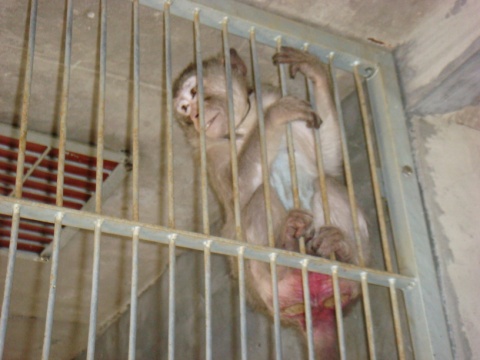 | | | |

| **H. Parental Behavior** | | | | | | | | | | | | |
| --- | --- | --- | --- | --- | --- | --- | --- | --- | --- | --- | --- | --- |
| **Behavior** | | | | Licking anus of infant | | | | | | | | |
| **Description** | | | | A mother licking the anus of her infant. | | | | | | | | |
| **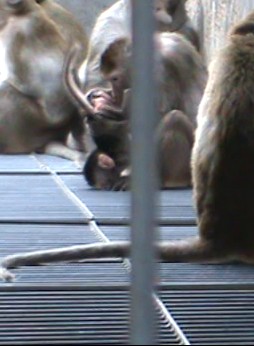** | | | | | | | | | | | | |
| **Behavior** | Checking anus of infant | | | | | | | | | | | |
| **Description** | A mother inspecting the anus of her infant by hand. | | | | | | | | | | | |
| 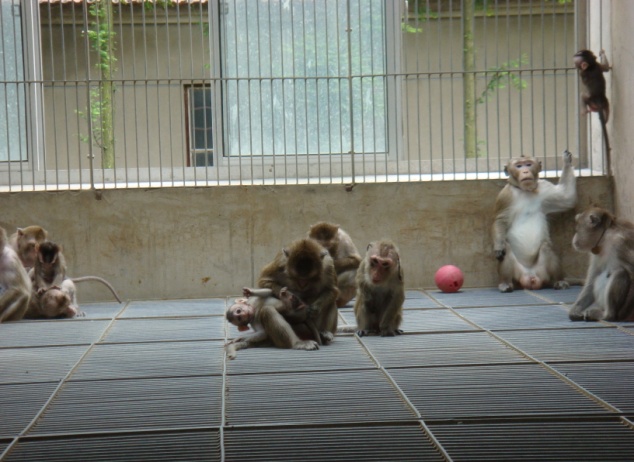 | | | | | | | | | | | | |
| **Behavior** | | | | | | | | | Nursing infant | | | |
| **Description** | | | | | | | | | A mother holding and nursing the infant. | | | |
| 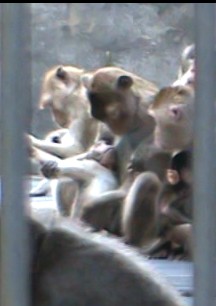 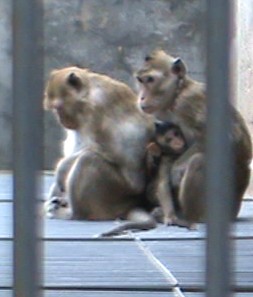 | | | | | | | | | | | | |
| **Behavior** | | | | | | | | | Defending infant | | | |
| **Description** | | | | | | | | | A mother protecting her infant from aggressor(s). | | | |
| 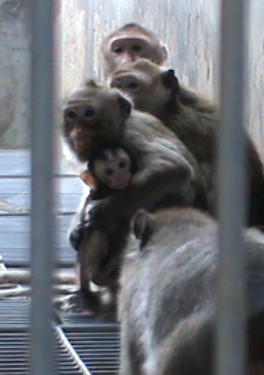 | | | | | | | | | | | | |
| **I. Amicable Behavior** | | | | | | | | | | | | |
| **Behavior** | | | | | | Grooming | | | | | | |
| **Description** | | | | | | Grooming another subject by hand (including combing the hair and/or catching pests). | | | | | | |
| 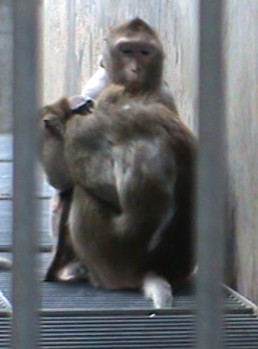 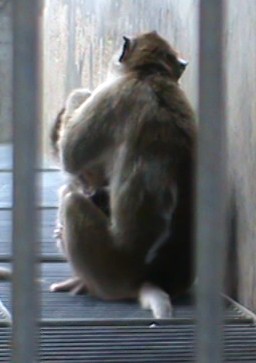 | | | | | | | | | | | | |
| **Behavior** | | | | | | Being groomed | | | | | | |
| **Description** | | | | | | Being groomed by another subject. | | | | | | |
| **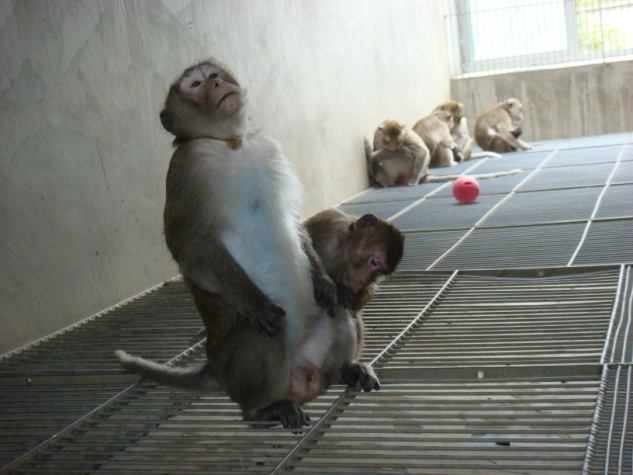** | | | | | | | | | | | | |
| **J. Conflict behavior** | | | | | | | | | | | | |
| **Behavior** | | | | | | | | Driving | | | | |
| **Description** | | | | | | | | Driving another subject away, usually by a higher-ranking subject. | | | | |
| **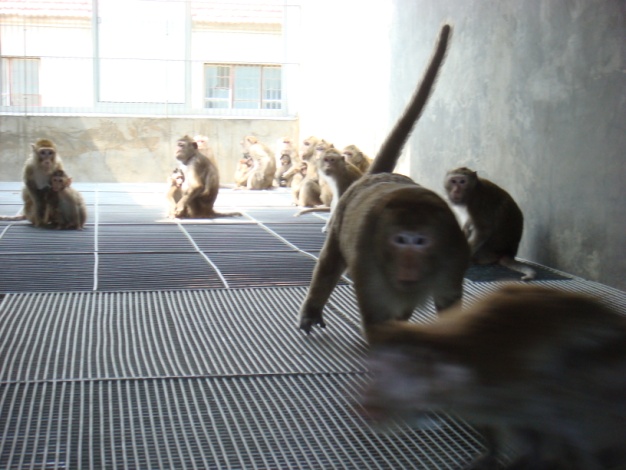** | | | | | | | | | | | | |
| **Behavior** | | Attacking | | | | | | | | | | |
| **Description** | | Attacking another subject (usually over territory, food, or mating). | | | | | | | | | | |
| 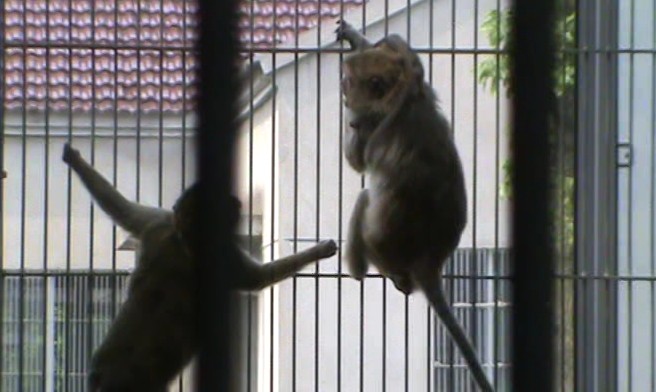 | | | | | | | | | | | | |
| **Behavior** | | Fleeing | | | | | | | | | | |
| **Description** | | Fleeing from a conflict with another subject. | | | | | | | | | | |
| 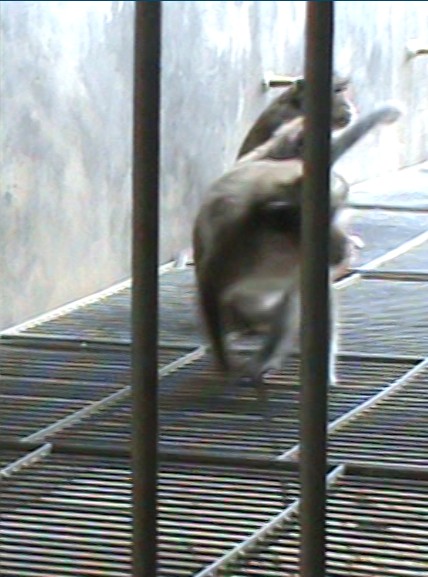 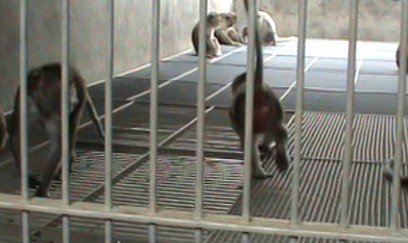 | | | | | | | | | | | | |
| **Behavior** | | Pulling foreleg | | | | | | | | | | |
| **Description** | | Pulling the foreleg of another subject. | | | | | | | | | | |
| 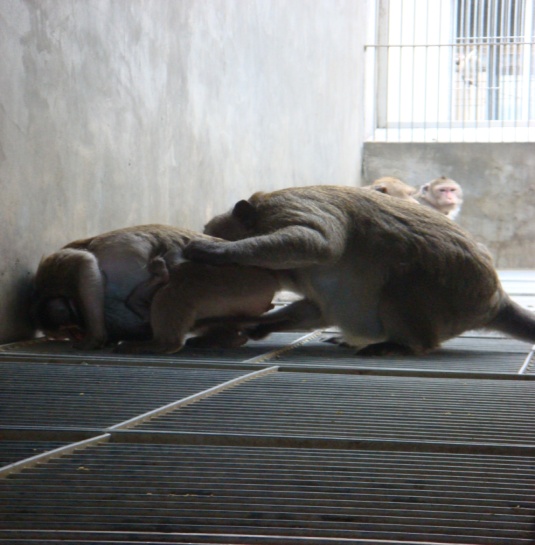 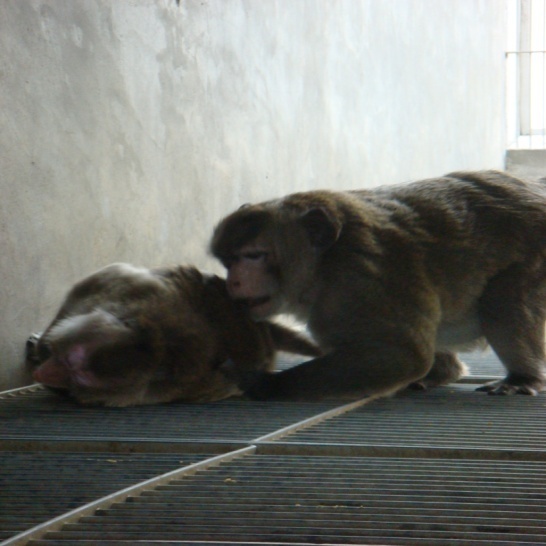 | | | | | | | | | | | | |
| **Behavior** | | | | | | | | | | Threatening | | |
| **Description** | | | | | | | | | | Threatening another subject (usually by opening the mouth and bearing the teeth and/or staring). | | |
| 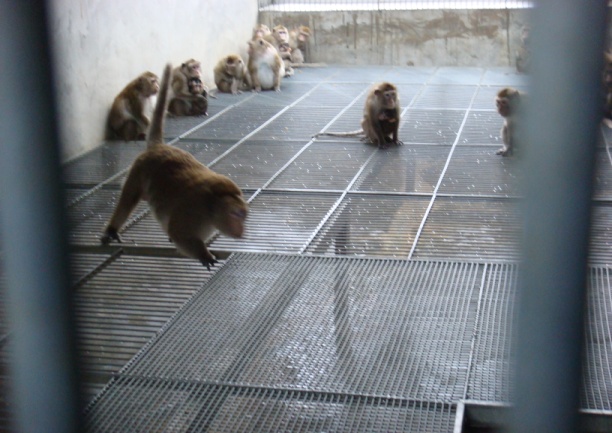 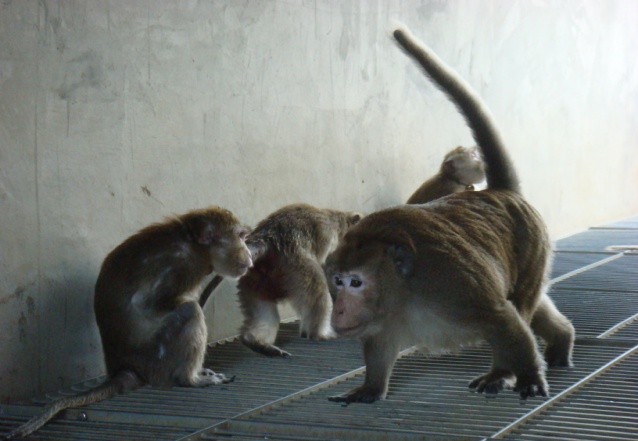  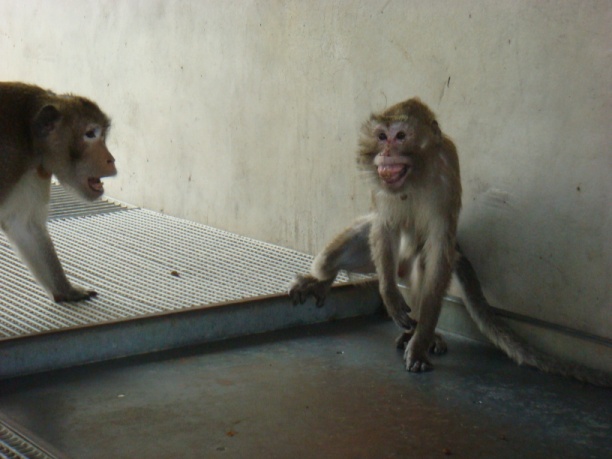 | | | | | | | | | | | | |
| **Behavior** | | | | Biting | | | | | | | | |
| **Description** | | | | Biting another subject with the teeth. | | | | | | | | |
| 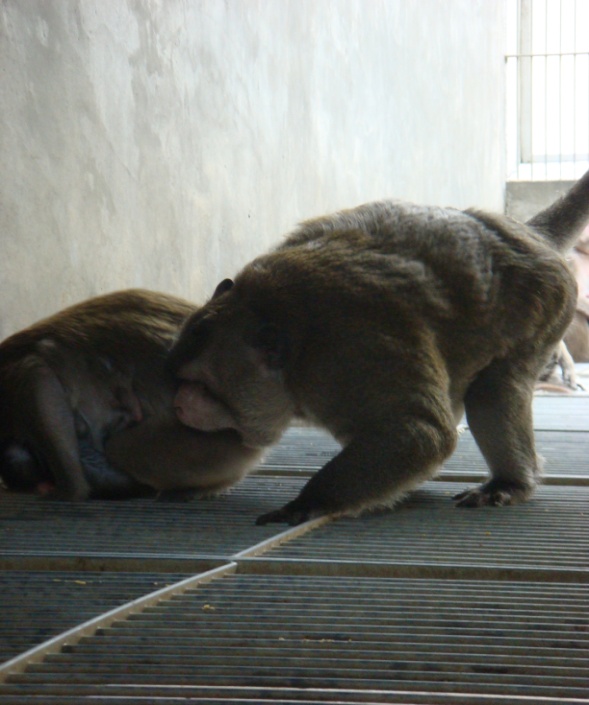 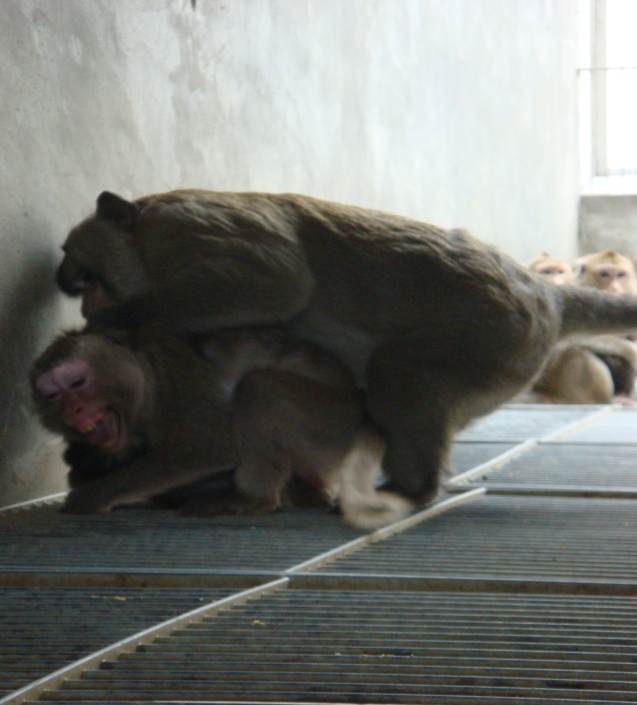 | | | | | | | | | | | | |
| **K. Vigilance behavior** | | | | | | | | | | | | |
| **Behavior** | | | | | Watching company | | | | | | | |
| **Description** | | | | | Watching other subjects. | | | | | | | |
| 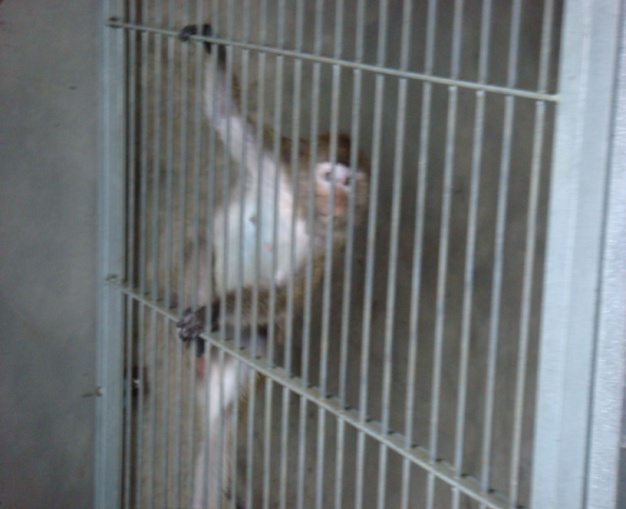 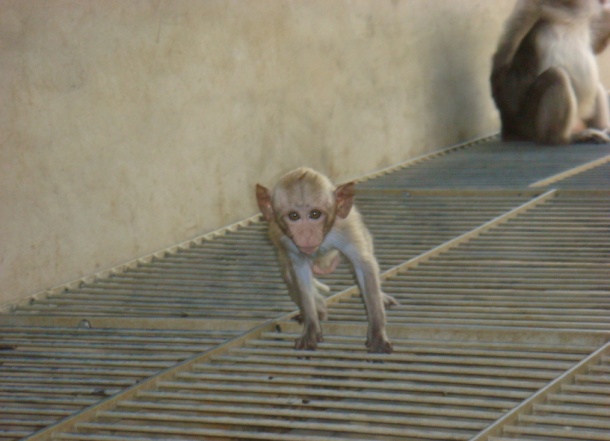 | | | | | | | | | | | | |
| **Behavior** | | | | | | Alarmed jumping | | | | | | |
| **Description** | | | | | | Jumping in alarm from the floor in response to a threat. | | | | | | |
| 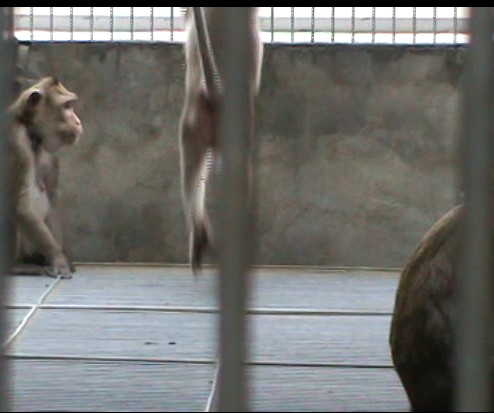 | | | | | | | | | | | | |
| **L. Locomotion behavior** | | | | | | | | | | | | |
| **Behavior** | | | | | | | Quadrupedal walking on floor | | | | | |
| **Description** | | | | | | | Walking on the floor on all four limbs. | | | | | |
| 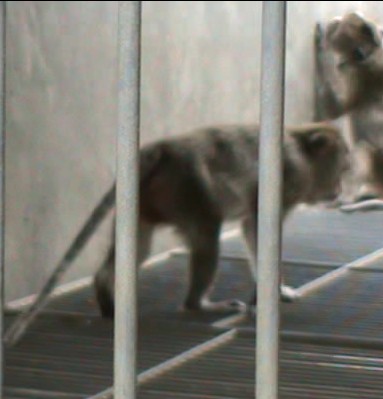 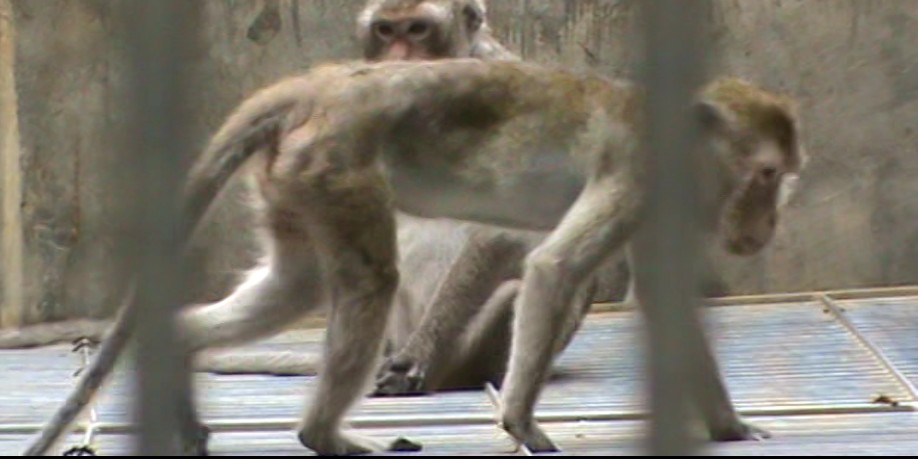 | | | | | | | | | | | | |
| **Behavior** | | | | | | | Galloping | | | | | |
| **Definition** | | | | | | | Galloping (usually to avoid conflict). | | | | | |
| **M. Communication behavior** | | | | | | | | | | | | |
| **Behavior** | | | Lip smacking | | | | | | | | |  |
| **Description** | | | Smacking of the lips to communicate with other subject(s). | | | | | | | | | |
| 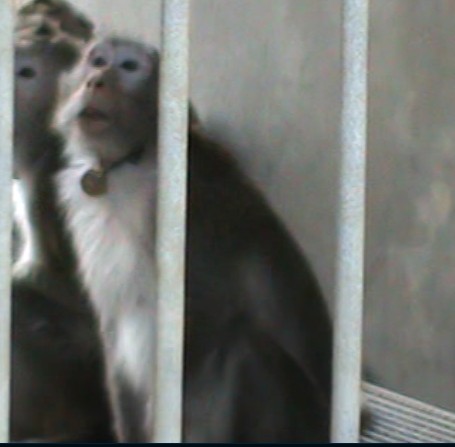 | | | | | | | | | | | | |
| **N. Miscellaneous Behavior** | | | | | | | | | | | | |
| **Behavior** | | | Playing | | | | | | | | | |
| **Description** | | | Playing with a toy or infant. | | | | | | | | | |
| 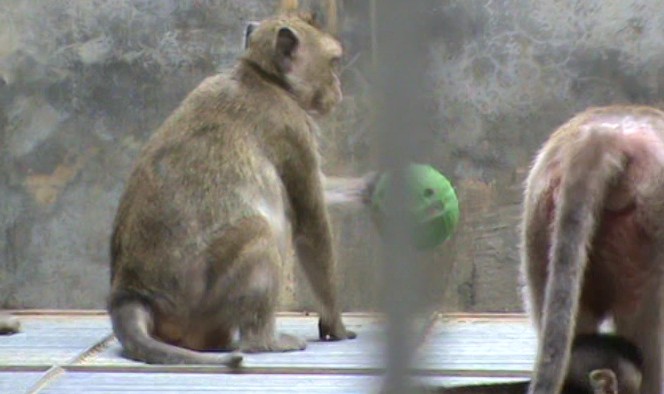 | | | | | | | | | | | | |
| **Behavior** | | | | | | | | Scratching by hind leg | | | | |
| **Description** | | | | | | | | Self-scratching any portion of the body by the hind limb. | | | | |
| 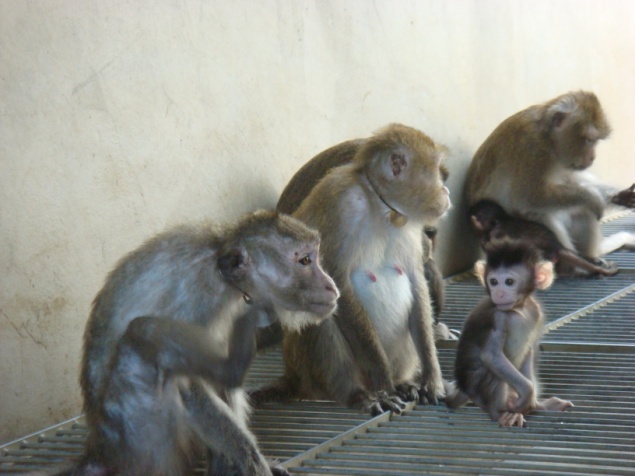 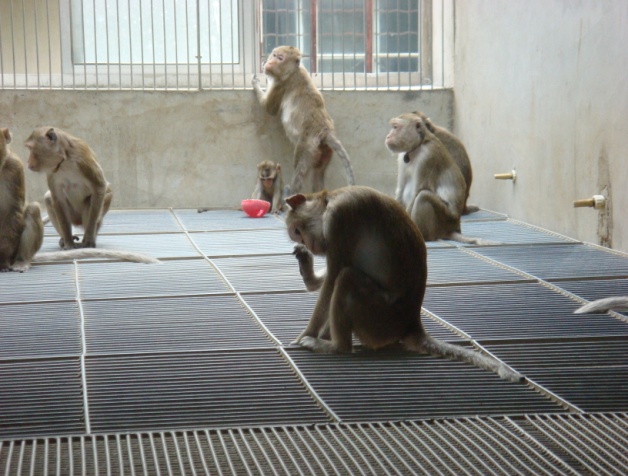 | | | | | | | | | | | | |
| **Behavior** | | | | | | | | | | | Scratching by foreleg | |
| **Description** | | | | | | | | | | | Self-scratching any portion of the body by the forelimb. | |
| 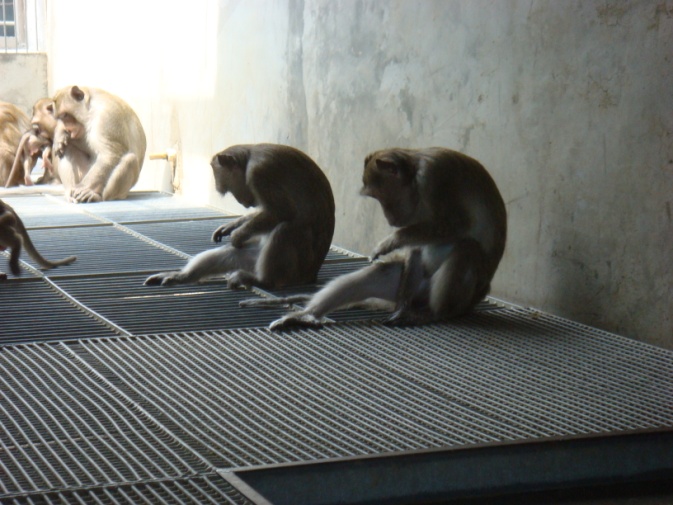 | | | | | | | | | | | | |
